# Review of Some of the Surgical Cases Which Have Lately Occurred in the Royal Infirmary of Edinburgh. A Clinical Lecture Delivered to the Students of Surgery in That Institution, on Thursday, 26th February, 1829

**Published:** 1829-08

**Authors:** George Ballingall

**Affiliations:** Fellow of the Royal College of Surgeons, Surgeon Extraordinary to the King, Regius Professor of Military Surgery in the University of Edinburgh, and one of the Surgeons to the Royal Infirmary.


### DR. BALLINGALL'S CLINICAL LECTURE.

*Review of some of the Surgical Cases which have lately occurred in the* ROYAL INFIRMARY *of* EDINBURGH. *A Clinical Lecture delivered to the Students of Surgery in that Institution, on Thursday, 26th February,* 1829, *by* GEORGE BALLINGALL, M.D. F.R.S.E.; Fellow of the Royal College of Surgeons, Surgeon Extraordinary to the King, Regius Professor of Military Surgery in the University of Edinburgh, and one of the Surgeons to the Royal Infirmary.

(Continued from p. 32.)

AMONG the cases of secondary amputation, I would notice that of Helen Coghill, æt. twenty-one, as affording an example of the severe sufferings occasionally experienced from inflammation of the joints, and as affording room for a single remark on the mode of amputating the thigh.

This poor girl was admitted on the 8th of October, and the following report entered on the journal:

"The left knee is swelled, hot, and painful; the patella is moveable, but the slightest motion or pressure upon this bone is attended with very severe pain in the condyles of the femur. The limb is kept constantly in the extended posture. Pulse natural; tongue clean; belly regular. States that her knee was first inflamed ten years ago, in consequence of a fall, from the effects of which she never altogether recovered. Eighteen months ago, after a similar accident, the inflammation was much aggravated, and has been more severe than ever during the last seven weeks.—Applic. Hirud. xx. genu."

Subsequent to this, various remedies were employed, but without any very obvious or permanent relief, and on the 3d of November I find the following report entered in the journal:

"Took three grains of opium, and slept a little; pulse 124; tongue slightly furred; no sweating; bowels regular; appetite rather worse. Previous to her admission she had been cupped, and leeches had been applied with benefit. Since her admission she has been leeched three times, and cupped three times to a large amount, and has also had a blister applied; but the disease has certainly become worse."

Leeches were again applied, and were followed at a short interval by the use of moxas on either side of the patella; but none of these remedies seemed to check the progress of the disease. The pain was so exquisite upon any attempt to move the limb, as to preclude any satisfactory examination of the joint; her appetite failed; she was occasionally distressed with nausea and retching, and her bowels became greatly disordered. In short, her sufferings were so severe as to induce her to seek relief by the removal of the limb; but, when this was seriously proposed to her, she requested permission to consult her friends on the subject, and it was not until the 5th of December that she made up her mind to the operation, which was immediately performed. She took seventy drops of laudanum, and slept a little on the following night.

For some days after this she was harassed with nausea and occasional retching, her pulse varying from 136 to 140; but her bowels became more regular, and her general appearance improved. She was distressed with some superficial ulcerations on the back, but the stump from the first assumed a very promising appearance; the greater part of it united by the first intention, and indeed I am inclined to say that, upon the whole, it healed too rapidly: that is to say, that union had taken place between the lips of the wound before the inflammation contiguous to it had sufficiently abated. In consequence of this, some small phlegmonous swellings formed and burst in the line of the cicatrix, after she left the hospital, which she did on the 3d of January.

On laying open the joint after the amputation of the limb, the disease in this poor girl's knee was found to have made very extensive ravages. The preparation, which I now again exhibit to you, shows that the cartilages covering all the bones of the joint, with the exception of that on the inner condyle of the femur, were either entirely destroyed or floating loose, being completely detached from the bones.

You would observe, gentlemen, that, in amputating this young woman's thigh, instead of forming two lateral flaps, as I had hitherto been accustomed to do, I formed an anterior and posterior flap, the one from the extensors on the fore part, and the other from the flexors on the back part of the thigh. This is a mode of operating which I first saw practised by Mr. Liston in the cases of two boys whom he operated upon in the house last autumn; and in the case of such young subjects, who cannot readily be made to retain their stumps in a desirable position, but are constantly inclined to elevate the point of the stump, it appears to me to offer decided advantages: in the first place, it obviates the projection of the bone between the lateral flaps, which I am told has sometimes occurred; and, in the next place, you will see that, the more the point of the stump is elevated, the more are the extensor muscles relaxed, so as to afford a covering for the point of the bone. Other collateral advantages attendant upon this mode of forming the flaps are pointed out by Mr. Creasers, formerly of the Bath Infirmary, in the twenty-second volume of the Edinburgh Medical and Surgical Journal; although he, indeed, recommends the flap to be formed by cutting from the surface towards the bone, instead of transfixing the limb and cutting outwards.

The plan of dressing stumps after the common circular amputation of the thigh, so as to place the line of the cicatrix transversely, instead of perpendicularly, you will find advocated both by Mr. Guthrie and by Mr. Copland

Hutchison; the latter of whom, in the first edition of his surgical work, gave a marginal sketch well calculated to illustrate his valuable remarks upon this point.

The only other case of amputation with which I propose to detain you at present is that of John Browne, æt. thirty-seven, a seaman, who, about twelve months previous to his admission, had been cast away in a vessel upon the coast of Norway, and had remained five days on the wreck. The circumstances under which he was admitted are as follows: "Has lost all his toes in consequence of frostbite. On the under surface of the left foot there is an extensive ulcer, which, from the very extensive destruction of the skin which has taken place, scarcely appears capable of cicatrization.

"States that his toes sloughed off during exposure to cold on the coast of Norway in November last, and that the ulcer of the left foot has never been cicatrized."

On examining accurately the state of this poor man's foot, it was obvious that no firm or permanent cicatrix was likely to be formed without sacrificing the metatarsal bones, and accordingly the following operation was determined on, which I executed on the 4th of November.

"An incision, convex anteriorly, having been made from a little beyond the base of the first metatarsal bone, to the most extreme point of the base of the fifth, a flap was formed from the integuments on the dorsum of the foot. The four outer metatarsal bones were then disarticulated, the internal cuneiform bone sawn through, and a corresponding flap formed from the integuments of the sole; the two flaps were then united by three points of the interrupted suture."

The dressings were removed on the 8th, when "the wound was found partially united; at one point the integuments had sloughed for about the extent of a shilling." Subsequent to this, he sustained a severe attack of erysipelas, which, in two or three points contiguous to the wound, terminated in the formation of matter under the skin; it assumed the erratic form over the other parts of his limb, and encroached slightly upon the trunk of his body. The cure was, upon the whole, tedious; but he ultimately obtained a good stump, and was well satisfied with the operation. He remained in hospital until the 5th of January, when a passage was procured for him to London, where he expected something to be done for him by the owners of the vessel in whose service he had been so severely mutilated.

The only circumstance worthy of notice in this operation is that, in consequence of the destruction of soft parts on the anterior part of the sole, I was obliged to form a longer flap from the dorsum of the foot than what I think desirable. Had circumstances admitted of it, I should have preferred forming a single large flap from the sole, and turning it up over the anterior extremities of the tarsal bones; thus bringing the cicatrix up towards the dorsum of the foot, and rendering it less liable to be injured in walking.

After these remarks upon amputation, I would now solicit your attention to a very interesting case of severe injury of the ankle, with exposure of the astragalus, and consecutive luxation of the joint; a case in which it was apprehended that amputation might have been necessary, but which ultimately did well under Dr. Hunter's care, by the use of repeated bleedings.

Martin M'Owen, æt. nineteen, admitted 19th October.—"Situated over the fore part of the ankle is a contused wound, the size of the palm of the hand, occasioned by a loaded waggon passing over it. The astragalus is felt quite bare on the outer side. There does not appear to be any fracture. The limb was placed on M'Intyre's splint, and a poultice applied.

"October 20th.—Was bled twice during the course of yesterday. Little swelling of limb. There appeared to be a dislocation of the ankle, which was easily reduced. Slept badly. Bowels freely opened; tongue moist, pulse sixty, skin hot, no thirst.—Venæsectio ad ℥xij. Cataplasma ferment. Sol. Tart. Antim. ℥ss. every hour.

"21st.—Blood buffed and cupped; some sleep; tongue clean and moist; belly open; pulse eighty-four. Skin cool; some thirst; little swelling of leg, which is looking well.—Venæsectio ad ℥xij. Sulph. Magnes. ℥i. statim. Cont. Sol. Tart. Antimon.

"25th.—Sloughs nearly all separated; pulse seventy-eight, rather sharp; belly open: tongue moist.—Venæsectio ad ℥xij. Tart. Antim. gr. ij.; Aquæ ℥viij. M. capiat ℥i. tertiâ quâque horâ.

"26th.—Blood buffed and cupped; slept badly from pain in limb; no swelling; discharge increased, and not so healthy; belly open; tongue moist.—Venæsectio ad ℥x.

"27th.—Granulations more healthy; no pain of leg; slept well; belly open, tongue moist; pulse eighty-four.

"November 1st.—Leg looking well. Was ordered six ounces of beefsteak and a pint of porter daily; and on the 2d of January was dismissed cured."

Amongst the cases of chronic diseases of the bones, you had two very remarkable instances of the separation of extensive portions of the cranium in two boys who were under Mr. Liston's care, during the present course. The first of these, James Thom, æt. thirteen, was admitted on the 10th of December, and stated "that, two and a half years ago, he received a blow upon the head from a stone; a swelling formed, which opened three days after the accident; and a small quantity of blood was evacuated, since which time several portions of bone have exfoliated. There is an opening immediately over the junction of the parietal bones, towards their back part; a small bridge of integument divided the opening; the matter in the sore can be distinctly seen rising and falling, corresponding with the pulsation of brain. On introducing a probe, the bone is felt bare for a considerable distance round the wound on all sides. His health has not suffered, neither has he been at any time confined to bed; no pain of head; pupils appear dilated, but are perfectly sensible; tongue white, appetite good; pulse seventy-two, somewhat irregular.

"December 14th.—An incision was made in the scalp, and the bone found extensively bare. No bad symptoms since."

On the 26th, it is stated that "a circular portion of bone was removed, to give a free exit to the matter; has had no headach; slept well; tongue clean." From this period the wound assumed a healthy aspect, the patient had no headach nor other unpleasant symptom; and about the middle of January he was dismissed, with instructions to return and show himself occasionally. Upon the last examination, the wound was cicatrizing rapidly, and the boy's general health good.

Another case, of a more serious aspect, and attended with repeated convulsive attacks, occurred in the person of Henry Lee, æt. thirteen, who was admitted on the 22d of December, and whose case is thus detailed in the journal:

"Received a blow on his head twelve months ago; a swelling formed, which was opened a few days after the accident. Had a good deal of pain in his head after the abscess was opened, and about a week after this had a fit, which was followed by a discharge of matter, and relieved the pain of the head. Has had a great number of fits, which have all been followed by a discharge of pus; has had no fit since the 17th September last. There is an opening, the size of a shilling, in the scalp, situated over the junction of the occipital bone with the parietal bones and the sagittal suture; the bone is black and bare for a considerable distance round; the discharge is thin and offensive; has no pain of head; health and appetite good; pupil dilated, but perfectly sensible; tongue moist; pulse 104.

"There is a fistulous opening over the sternum, leading down to the bone, which is bare; an abscess formed about the same time with that on the head, and was opened. No pieces of bone have been discharged.

"December 23d.—A crucial incision was made, and a piece of bone, the whole thickness of the cranium, was found loose, and removed." Had no bad symptom after the operation. On the 27th he was ordered animal food, and a few days afterwards four ounces of wine daily. From this period his cure went on progressively, and on the 10th of January he was made an outpatient. The fistulous opening over the sternum was touched with the potential cautery previous to his leaving the house, and has since healed.

Both these cases afforded examples of the beneficial interference of art in removing large portions of the cranial bones when in a state of disease. The contrast between such cases and those of recent injury is very remarkable; for, while in the latter every experienced surgeon dreads the extensive exposure of the dura mater, yet he knows that, in cases like those I have just detailed, where the dura mater has been for some time detached from the interior of the skull, and has become covered with granulations, the carious or necrosed bone may be removed with freedom, and often with advantage: witness, amongst many other cases, the very remarkable one detailed by Saviard, of a woman in the Hôtel Dieu at Paris, "who underwent successive exfoliations of the cranial bones to such an extent that the pieces, when put together, resembled the skullcap as it is sawn off in dissections."

In the case of another patient of Mr. Liston's, John Alison, æt. twenty-one, who was admitted on the 27th October, an instance was seen of the successful removal of a portion of diseased bone from the os calcis, a bone of very different texture and situation. The detail of this case given in the journal is as follows:

"States that nine years ago he sprained his left ankle. There have been at different times sores formed, which healed up without any bone being discharged. Ten weeks ago received a slight injury, when it again swelled, and several abscesses formed and burst: one small piece of bone was discharged. There are three sores situated on the outer, and the same number on the internal malleolus.

On probing the sores on the outer side, the os calcis is found carious, and the probe appears to enter the joint. The bones opposite intersected on all sides by sinuses. Health and appetite good at present. Motion of the joint not impaired."

On the 3d of November, "a considerable portion of the os calcis was removed by Mr. Liston, and the cavity stuffed with lint."

Very little pain or swelling succeeded to the operation; a poultice was applied over the dressings. On the 13th, a seton was drawn through the diseased part of the foot; and on the 4th of December it was reported that "no dead piece of bone can now be felt; granulations healthy; general health good."

About the beginning of January, the sores were nearly all healed. Some soft swelling remained about the ankle, the motions of which were perfect. It was done up with plaster and bandage, in the manner recommended by Mr. Scott, of the London Hospital, and the patient dismissed.

Akin to the last-mentioned case is that of Alexander Beveridge, æt. fifteen, admitted on the 25th of November.

"States that eighteen months ago a swelling began to form over the left os calcis on the inner side. This broke, and discharged a quantity of matter. Two small pieces of bone have been discharged. There is a fistulous opening below the malleolus, leading down to the bone, which is bare; the motions of the joint are perfect; health good."

On the 28th it is reported, that "a large portion of dead bone was removed yesterday by Mr. Liston, with the assistance of the trepan; slept badly; some headach; pulse 180; skin rather hot; tongue clean; bowels open; no swelling of limb."

On the 2d of December, "dressings were removed; sore looking well; health good."

From this time forward I find nothing of any importance noted in the journal; the patient's health continued good under the use of animal food and porter, the sore granulated kindly, and on the 14th of January he was dismissed cured.

These cases, gentlemen, I have been induced to bring to your recollection, because, although they offer nothing very striking in the detail, they are well calculated, in my opinion, to encourage a more successful practice in a class of cases which have frequently been (if I may use the expression,) slurred over, and have too often been allowed to go on from bad to worse, until the patient's life has been brought into hazard, and has perhaps been ultimately saved only at the expense of his limb.

Of the partial excision of bones in cases of carious joints, I have, during the present winter, seen some very remarkable and successful examples in the practice of Mr. Syme, which are, I believe, intended to be laid before the profession * These cases have struck me so forcibly, that I begin to be almost ashamed to look my own preparations in the face. The museum of the Royal College of Surgeons will, I am afraid, bear witness that I, as well as other surgeons, have amputated several limbs which might have been saved by the excision of the carious joints.

[To be continued.]

### MIDWIFERY.

* Vide "Three Cases in which the Elbow-joint was successfully excised, with some general Observations on the Treatment of Caries. By James Syme, Esq. Surgeon, and Lecturer on Surgery in Edinburgh." Edinb. Med. and Surg. Journal, April 1829.—Editors.

